# Highly Virulent Getah Virus Subgroup GIII‐c Emerging in Pigs in China

**DOI:** 10.1155/tbed/3201005

**Published:** 2026-08-21

**Authors:** Zhaoying Xiang, Xingchen Wang, Yuchen Huang, Xinyu Gao, Lei Zhou, Xinna Ge, Jun Han, Yongning Zhang, Hanchun Yang

**Affiliations:** ^1^ State Key Laboratory of Veterinary Public Health and Safety, College of Veterinary Medicine, China Agricultural University, Beijing 100193, China, cau.edu.cn; ^2^ Key Laboratory of Animal Epidemiology of Ministry of Agriculture and Rural Affairs, College of Veterinary Medicine, China Agricultural University, Beijing 100193, China, cau.edu.cn; ^3^ Swine Farming Division, Guangdong Haid Group Co., Ltd., Haida Science Park, Guangzhou 511400, China

**Keywords:** contact transmission, Getah virus, pathogenicity, phylogenetic analysis, subgroup GⅢ-c

## Abstract

Getah virus (GETV) is an emerging mosquito‐borne zoonotic alphavirus that poses a threat to multiple animal species, particularly pigs, and carries significant public health implications. Nevertheless, the molecular evolution and pathogenicity of circulating GETV strains in China remain incompletely defined. In this study, three GETV strains were isolated from diseased piglets during concurrent outbreaks on three geographically distinct commercial pig farms in China in August 2025. The isolates shared 99.67%–99.91% genomic nucleotide (nt) identity and were phylogenetically classified into subgroup GⅢ‐c. Amino acid sequence alignment against 136 reference strains from the GenBank database identified four substitutions in Nsp1 and Nsp3 that were absent from all reference strains, along with six conserved residues largely concentrated in a distinct subclade formed by the three isolates and five related reference strains. Pathogenicity evaluation using a representative isolate (GETV‐YY/2025) in 7‐day‐old piglets revealed that the infected animals developed severe clinical signs, including fever, diarrhea, ataxia, and hindlimb paralysis, with a high mortality rate of 77.8% (7/9). The strain exhibited broad tissue tropism, with high viral loads detected in pharyngeal and rectal swabs, serum, and multiple visceral organs as early as 1 day postinoculation, accompanied by obvious cerebral vascular congestion and pulmonary hemorrhage. Notably, efficient contact transmission to sentinel piglets was observed, resulting in one fatality among the three contact animals (33.3%). Collectively, these findings characterize the emergence of a highly virulent GETV subgroup GⅢ‐c circulating in Chinese pig populations, highlighting its capacity for efficient contact transmission and severe pathogenesis in neonatal pigs. This study provides critical insights into the evolutionary dynamics of GETV and informs the urgent development of prevention and control strategies against this re‐emerging veterinary pathogen.

## 1. Introduction

Getah virus (GETV), a member of the *Alphavirus* genus within the Togaviridae family [1], is an arbovirus capable of infecting a broad range of vertebrate hosts, including horses [2], pigs [3], cattle [4], blue foxes [5], and red pandas [6]. Notably, the detection of anti‐GETV antibodies in febrile patients suggests a potential zoonotic risk [7]. The clinical manifestations of GETV infection vary considerably across host species. Infected horses typically develop mild symptoms, such as fever, rash, and limb edema, with low mortality rates [8, 9]. In adult pigs, infection is often subclinical; however, it can cause abortion and fetal death in pregnant sows [10–12]. In contrast, GETV infection in piglets frequently results in severe disease characterized by fever, depression, diarrhea, tremors, and high mortality [11–13]. GETV has been reported in several Asian countries, including China, Japan, South Korea, Malaysia, and Mongolia [14]. In China, the frequency of GETV outbreaks has markedly increased since 2018, posing a growing threat to the swine industry and raising public health concerns [4, 12, 15–18]. Currently, no commercially available vaccines exist for GETV, underscoring the importance of fundamental research on this pathogen. Consequently, the isolation and characterization of circulating strains, along with analyses of their evolution and pathogenicity, are essential for the risk assessment and the development of future control strategies.

The GETV genome is a single‐stranded, positive‐sense RNA of ~11.7 kb [1], comprising a 5′‐untranslated region (UTR), a 3′ poly‐A tail, and two open reading frames that encode four nonstructural proteins (Nsp1−4) and five structural proteins (C, E3, E2, 6 K, and E1). Nsp1 is involved in RNA capping and membrane anchoring of the replication complex [19], whereas Nsp3 plays critical roles in viral replication and host adaptation [20]. Thus, mutations in these proteins can influence viral fitness and virulence [21]. The E2 glycoprotein serves as a major antigenic determinant, participating in receptor binding and viral entry, making it a key target for phylogenetic studies [16]. Therefore, systematic comparisons of amino acid variations in these proteins among contemporary GETV isolates, especially those derived from concurrent outbreaks, are warranted.

Current knowledge of GETV pathogenicity in piglets has revealed several key features [21–24]: the virus exhibits broad tissue tropism, replicating across multiple organs; it causes severe pathological lesions, particularly vascular congestion in the brain and pulmonary hemorrhage; and it can be shed in feces. Furthermore, mortality rates vary among the different GETV strains. Nevertheless, critical aspects such as the detailed in vivo replication dynamics and the potential for contact transmission remain poorly understood. In this study, we isolated three GETV strains from clinical samples collected on three commercial pig farms in China. These isolates were characterized through whole‐genome sequencing, phylogenetic analysis, and detailed amino acid comparisons with 136 reference strains, in addition to pathogenicity assessment in piglets. This comprehensive characterization sheds light on the genetic evolution and pathogenicity of contemporary GETV strains, providing a scientific foundation for understanding this emerging pathogen.

## 2. Materials and Methods

### 2.1. Cells and Antibody

Vero cells (ATCC CCL81) were cultured in Dulbecco’s modified Eagle’s medium (DMEM; 12491015, Gibco, USA) supplemented with 10% fetal bovine serum (FBS; 16140071, Gibco, USA) and 1% penicillin–streptomycin (15140122, Gibco, USA) at 37°C with 5% CO_2_. A monoclonal antibody (mAb), designated 3G9, specific to the GETV E2 protein, was generated in our laboratory.

### 2.2. Sample Collection

In August 2025, clinical samples suspected of GETV infection were collected from three commercial pig farms in China. The samples comprised intestinal tissues, lymph nodes, and sera obtained from diseased piglets. Specifically, five intestinal tissue samples and one lymph node sample were collected from Pig Farm 1 located in Shaoguan city, Guangdong province; four intestinal tissue samples and one lymph node sample were collected from Pig Farm 2 located in Yueyang city, Hunan province; and 10 serum samples were collected from Pig Farm 3 located in Chenzhou city, Hunan province.

### 2.3. RNA Extraction and Quantitative Real‐Time PCR (qRT‐PCR)

All sample processing, virus isolation, and cell culture procedures were conducted in a biosafety level 2 laboratory. For tissue samples, approximately soybean‐sized blocks were excised from the interface between lesioned and normal tissues, and 200 mg of each sample was homogenized in phosphate‐buffered saline (PBS). The homogenates were then centrifuged at 8000 × *g* for 15 min at 4°C, and the resulting supernatants were collected and stored at −80°C until use. Total RNA was extracted from 200 μL of tissue homogenate supernatant, serum, or swab samples using the TRIzol reagent (R4801‐01, Magen, China) according to the manufacturer’s instructions. First‐strand cDNA was synthesized using the FastKing RT Kit (KR116, TIANGEN, China) following the manufacturer’s instructions. GETV was detected by qRT‐PCR targeting the *Nsp2* gene, using specific primers and a TaqMan probe (forward: 5′‐GCAACTGCAAATCGACTATCGT‐3′; reverse: 5′‐TGTCAGACCCTGGGAAGCA‐3′; probe: 5′‐FAM‐ACGAGGTGATGACCGC‐MGB/NFQ‐3′) as described previously [25]. The qRT‐PCR assays were performed using FastFire qPCR PreMix (FP208, TIANGEN, China) in accordance with the manufacturer’s protocols.

### 2.4. Virus Isolation

To isolate the virus, two lymph node samples and one serum sample were collected from three distinct farms. Each tissue sample was homogenized in DMEM and centrifuged at 8000 × *g* for 15 min at 4°C. The resulting supernatant, along with the serum sample, was filtered through a 0.22‐μm syringe filter and inoculated onto monolayers of Vero cells. The cytopathic effect (CPE) was monitored every 24 h postinoculation. Upon observation of the visible CPE, the culture supernatant was harvested and subjected to blind passages. This process was repeated for three consecutive passages. The supernatant from the third passage (P3) was aliquoted and stored at −80°C for subsequent downstream experiments.

### 2.5. Indirect Immunofluorescence Assay (IFA)

Mock‐ and GETV‐infected Vero cells were fixed with ice‐cold absolute ethanol for 10 min, followed by incubation with mAb 3G9 at 37°C for 1 h. The cells were then washed three times with PBS and subsequently incubated with a fluorescein isothiocyanate (FITC)‐conjugated goat anti‐mouse IgG secondary antibody (AB_2338590, Jackson ImmunoResearch Laboratories, USA) at 37°C for 1 h. After three additional washes with PBS, the cell nuclei were counterstained with 4’,6‐diamidino‐2‐phenylindole solution (DAPI; 28718‐90‐3, Solarbio, China) at room temperature for 10 min. Specific fluorescence signals were visualized under a Nikon Eclipse Ti‐U fluorescence microscope.

### 2.6. Median Tissue Culture Infective Dose (TCID_50_) Assay

Vero cells were seeded in 96‐well plates and cultured until reaching 80%–90% confluence. The cell monolayers were washed gently with PBS, followed by inoculation with 100 μL/well of a 10‐fold serially diluted virus supernatant (dilutions ranging from 10^−1^ to 10^−7^) prepared in maintenance medium (DMEM supplemented with 2% FBS). The plates were incubated at 37°C in a 5% CO_2_ atmosphere for 48 h. Subsequently, an IFA was performed to detect viral antigen expression. Wells exhibiting specific fluorescence were recorded as positive, and the TCID_50_ value was calculated using the Reed–Muench method.

### 2.7. Determination of GETV Growth Kinetics

Confluent Vero cell monolayers grown in 24‐well plates were infected with the P3 virus supernatant at a multiplicity of infection (MOI) of 0.1. Following 1‐h virus adsorption at 37°C in a 5% CO_2_ atmosphere, the inoculum was removed, and the culture medium was replaced with the maintenance medium. Infected cell cultures were harvested at 0, 12, 24, 36, 48, and 60 h postinfection (hpi). Viral titers in the harvested samples were determined using the TCID_50_ assay.

### 2.8. Whole‐Genome Sequencing and Characterization of GETV

Total RNA was extracted from the P3 virus supernatant using the TRIzol reagent. The 3’ end of the viral genome was reverse transcribed using the primer 4F and 3’ RACE adaptor (Table 1), whereas the remaining genomic fragments were reverse transcribed with random primers provided in the commercial kit (KR116, TIANGEN, China). Based on the reference genome of strain NM2022 (GenBank Accession Number PP537546), four pairs of gene‐specific primers (Table 1) were designed to amplify the full‐length viral genome via RT‐PCR. Among these, the primer pair 4F/4R was used to amplify the 3’ end, while the remaining three pairs covered the rest of the genome. The resulting PCR products were subjected to nanopore sequencing (Jiangsu Kangwei Century Biotechnology Co., Ltd., China), followed by de novo or reference‐guided sequence assembly.

**Table 1:** Primers used for RT‐PCR amplification of the full‐length GETV genome.

Primer names	Sequences (5′→3′)	Location^a^	Amplicon length (bp)
1F	ATGGCGGACGTGTGACATC	1–19	3710
1R	CGGCCTGCATCCATAGGTAACC	3689–3710	
2F	CTGGTCACAAGTTATCAGCAGTGC	3511–3534	3905
2R	GACAGCGTGGACATCGCTCTAGTG	7392–7415	
3F	GTACAGCGTGTAGGGTTGCAGAC	7196–7218	3666
3R	GTGACGGCAGGTGCATCAATCAC	10839–10861	
4F	CTCAAGTTGTCGAGGCCTTCGTC	10626–10648	1112
4R	GGATCCGGTACCTCTAGATCAGA	—	
3′ RACE adaptor	GGATCCGGTACCTCTAGATCAGATTTTTTTTTTTTTTTTTT	—	—

^a^Primer positions are referenced to the GETV NM2022 strain (GenBank Accession Number PP537546).

Phylogenetic analysis was performed using the assembled full‐length GETV genome sequence and the corresponding *E2* gene sequences, along with 136 GETV reference strains retrieved from the GenBank database. All phylogenetic reconstructions were conducted using MEGA‐X software (Version 10.2.6) with the maximum likelihood method and nodal support assessed by 1000 bootstrap replicates. Pairwise nucleotide (nt) distances were calculated using the p‐distance model implemented in MEGA‐X. Subgroup classification within group III was based on an integrated evaluation of phylogenetic topology, bootstrap support, and pairwise genetic distances of the *E2* gene. Phylogenetic nodes with bootstrap support ≥0.70 were considered candidate subgroups, within which further subdivision was assessed by examining pairwise genetic distances. Strains were assigned to independent subgroups if their minimum pairwise genetic distance to all other members of the same clade exceeded 0.02—a threshold selected based on the natural clustering pattern in the pairwise distance matrix, which clearly separated intrasubgroup (≤0.01) from intersubgroup distances. Amino acid sequences of the five structural proteins (C, E3, E2, 6 K, and E1) and four nonstructural proteins (Nsp1, Nsp2, Nsp3, and Nsp4) were aligned separately using MUSCLE within MEGA‐X. Residue variations were compared among the three isolates obtained in this study and the 136 reference strains to identify unique substitutions (present only in the isolates) and conserved substitutions (shared among the isolates but differing from the references).

### 2.9. Pathogenicity Analysis of GETV in Piglets

Prior to challenge, serum, nasopharyngeal swabs, and rectal swabs were collected from all sows and piglets and screened by qRT‐PCR to confirm the absence of common pathogens, including GETV, porcine reproductive and respiratory syndrome virus (PRRSV), and porcine epidemic diarrhea virus (PEDV). Seven‐day‐old piglets were randomly assigned to a challenge group and a mock control group (*n* = 12 per group) using a random number table. To minimize observer bias, this study was conducted in a blinded manner. Group allocation was carried out by an independent researcher who had no involvement in any subsequent procedures. All staff responsible for clinical monitoring, gross necropsy examinations, sample collection, and viral RNA quantification were kept unaware of the group assignment. Cages and samples were identified by code, and the allocation key was disclosed only after all evaluations were completed. In the challenge group, nine piglets were intramuscularly (i.m.) inoculated with 2 × 10^6^·^2^ TCID_50_ of the P3 GETV‐YY/2025 strain, while the remaining three piglets were housed as unchallenged sentinel contacts in the same pen without any physical separation, allowing continuous direct and indirect contact throughout the study under standard commercial farm conditions with a standard barn ventilation system. Mock‐control piglets received an i.m. injection of 2 mL of sterile physiological saline. All piglets were monitored daily for clinical signs and mortality, and surviving animals were euthanized at 12 days postinfection (dpi).

To assess viremia and viral shedding, serum samples, rectal swabs, and pharyngeal swabs were collected at 1, 3, and 5 dpi and analyzed by qRT‐PCR. In the mock control group, samples were collected from five randomly selected piglets at each time point. In the challenge group, samples were collected from all three sentinel piglets and from five randomly selected inoculated piglets at each time point. All piglets that died during the experiment or were euthanized at the endpoint (12 dpi) underwent gross pathological examination. Tissue specimens, including the brain, heart, liver, spleen, lungs, kidneys, intestines, mesenteric lymph nodes, mandibular lymph nodes, and inguinal lymph nodes, were collected. Samples were obtained from six of the seven inoculated piglets that died postchallenge, the two surviving inoculated piglets, all three sentinel piglets, and five randomly selected mock‐inoculated piglets. Viral RNA loads in swabs, serum, and tissues were quantified by qRT‐PCR following the same methodology described above.

### 2.10. Statistical Analysis

Data were analyzed using GraphPad Prism (Version 8.3) and are expressed as the mean ± standard deviation (SD) from at least three independent experiments. Viral growth curves were compared using two‐way analysis of variance (ANOVA), with time postinfection and viral isolate as the two independent factors. This was followed by Tukey’s multiple comparison test for pairwise comparisons between groups. A *p* value < 0.05 was considered statistically significant.

## 3. Results

### 3.1. Detection, Isolation, and Identification of GETV

**Figure 1:**
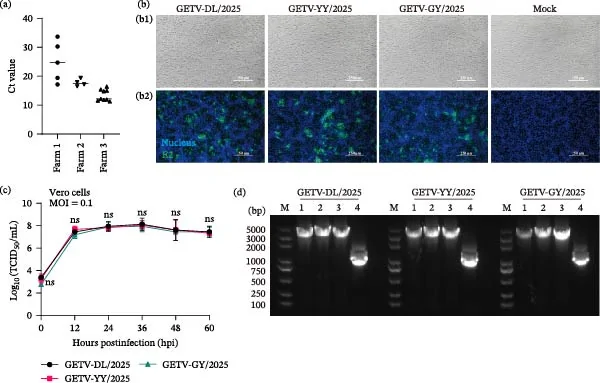
Detection, isolation, and identification of GETV. (a) Detection of GETV in clinical samples collected from three pig farms using qRT‐PCR. Each dot represents an individual sample, and horizontal bars indicate mean Ct values. (b) Cytopathic effects (b1) and immunofluorescence staining of the GETV E2 protein (b2) in Vero cells infected with the indicated isolates. The E2 protein was detected using the mAb 3G9, followed by FITC‐conjugated goat anti‐mouse IgG (green); cell nuclei were counterstained with DAPI (blue). (c) Multistep growth kinetics of the indicated isolates in Vero cells at an MOI of 0.1. Viral titers are shown as mean ± standard deviation from three independent experiments. Statistical significance was determined by two‐way ANOVA with Tukey’s multiple comparisons test; “ns” denotes no significant difference between any two groups in pairwise comparisons. Detailed pairwise comparison data are provided in Supporting Information 1: Table S2. (d) RT‐PCR amplification of the full‐length genomes of the indicated isolates.

In August 2025, outbreaks characterized by high mortality in piglets occurred on three commercial pig farms located in Guangdong and Hunan Provinces, China. The outbreaks resulted in the death of over 400 piglets, along with stillbirths or delivery of mummified fetuses in 26 pregnant sows. Affected piglets, aged 3–8 days, exhibited nasal tip cyanosis and neurological signs, including tremors and vocalizations (screaming), while a small proportion also developed diarrhea. Based on the clinical presentation suggestive of GETV infection, clinical samples were subjected to qRT‐PCR analysis. All samples tested positive for GETV, with cycle threshold (Ct) values ranging from 13.38 to 25.05, confirming active GETV infection (Figure 1a).

Supernatants from two lymph node samples and one serum sample collected from the three affected farms were inoculated onto Vero cells for virus isolation. Infected Vero cells developed a pronounced CPE, characterized by cell rounding and detachment (Figure 1b). The identity of the isolated viruses was further confirmed by IFA using mAb 3G9 specific to the GETV E2 protein. Strong specific fluorescence signals were observed in infected cells, whereas no fluorescence was detected in the mock‐infected control group (Figure 1b). The three isolates were designated as GETV‐DL/2025, GETV‐YY/2025, and GETV‐GY/2025. The replication kinetics of the three GETV isolates were evaluated in Vero cells at an MOI of 0.1. All isolates displayed comparable multistep growth kinetics, reaching peak viral titers of ~10^8^ TCID_50_/mL at 36 hpi (Figure 1c).

To obtain the full‐genome sequences of the isolates, RT‐PCR was performed using four pairs of overlapping primers designed to span the entire GETV genome. Four amplicons were successfully obtained for each isolate (Figure 1d), and subjected to Sanger sequencing, followed by sequence assembly. Excluding the poly (A) tails, the complete genomic sequences of all three isolates were determined to be 11,689 nt in length. The genome sequences have been deposited in the GenBank database under Accession Numbers PX926921 (GETV‐DL/2025), PX927507 (GETV‐YY/2025), and PX926920 (GETV‐GY/2025).

### 3.2. Phylogenetic and Amino Acid Variation Analysis of GETV Isolates

Phylogenetic analysis based on the GETV *E2* gene sequence classified group Ⅲ lineages into three distinct subgroups (GⅢ‐a, GⅢ‐b, and GⅢ‐c), as supported by the phylogenetic topology, bootstrap values, and pairwise genetic distances (Figure 2a). All three isolates obtained in this study clustered within subgroup GⅢ‐c and exhibited high *E2* gene nt identities with one another, ranging from 99.68% to 99.92%. Among the 136 GETV reference strains retrieved from the GenBank database, the isolates showed the highest nt identity (99.76%–100%) with the swine‐derived strain GETV‐JX‐CHN‐22‐P7 and the lowest identity (92.92%–93.18%) with the group I prototype strain GETV MM2021.

Figure 2Phylogenetic analysis based on the GETV *E2* gene sequence (a) and the full‐genome sequence (b). Phylogenetic trees were constructed using the maximum likelihood (ML) method implemented in MEGA X. Bootstrap values (percentages) from 1000 replicates are shown at the branch nodes. Isolates identified in this study are indicated by red stars. Within group Ⅲ, branches are color‐coded by subgroup: red for subgroup GⅢ‐a, blue for subgroup GⅢ‐b, and black for subgroup GⅢ‐c. The red dashed box in (b) highlights a distinct subclade comprising three isolates and the reference strains GETV JL, GETV JX CHN 22 P7, GETV HD, HNzmd‐BY1, and GETV CX7.

**Figure:**
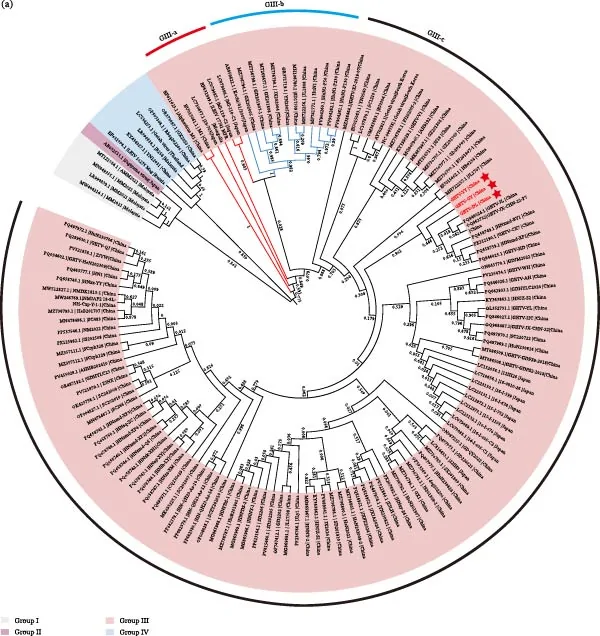

**Figure:**
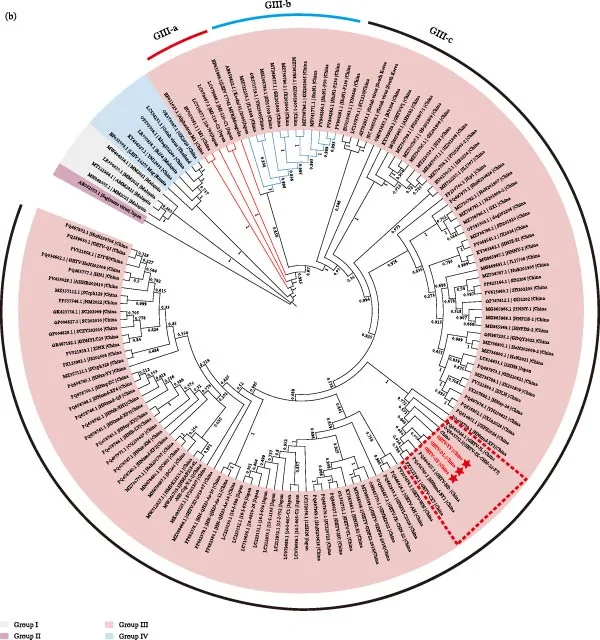

Consistent results were obtained from phylogenetic analysis based on complete genome sequences (Figure 2b). The full‐length genomes of GETV‐DL/2025, GETV‐GY/2025, and GETV‐YY/2025 were highly similar, sharing 99.67%–99.91% nt identity, and were likewise assigned to subgroup GⅢ‐c. Comparison with the 136 reference GETV genomes confirmed that these isolates were most closely related to GETV‐JX‐CHN‐22‐P7 (99.75%–99.79% identity) and most divergent from GETV MM2021 (93.98%–94.05% identity).

Amino acid sequence analysis of the five structural and four nonstructural proteins from the three isolates and 136 reference GETV strains revealed that most proteins were highly conserved, with only sporadic differences. However, pronounced variations were observed in Nsp1, Nsp3, Cap, and E2, where a total of 14 amino acid sites were identified (Table 2; the distribution of these residues among reference strains is provided in Supporting Information 2: Table S1). Among these, four substitutions were absent from all reference strains and thus represented novel variants in this dataset: Nsp1 K298Q (GETV‐GY/2025), Nsp1 A473T (GETV‐YY/2025), Nsp3 G62S (GETV‐DL/2025), and Nsp3 A447V (GETV‐YY/2025). Additionally, several positions were identical among the three isolates but exhibited variable frequencies in the reference strains, including Nsp1 T475 (34/136, 25%), Nsp3 I361 (12/136, 8.8%), Nsp3 V391 (6/136, 4.4%), Nsp3 E410 (5/136, 3.7%), Nsp3 T438 (11/136, 8.1%), and Nsp3 S443 (25/136, 18.4%). Notably, these six residues were largely concentrated within a distinct subclade that included GETV‐JL, GETV‐JX‐CHN‐22‐P7, GETV‐HD, HNzmd‐BY1, GETV‐CX7, and the three isolates from this study and were only sporadically present elsewhere in the phylogenetic tree outside this subclade (Supporting Information 2: Table S1 and Figure 2b). At the four positions where one isolate differed from the other two, two distinct patterns emerged. At Nsp1 position 306, Nsp1 position 427, and E2 position 51, the residue shared by two of the three isolates was also the predominant residue among the reference strains (present in ≥105 out of 136 strains). In contrast, at Cap position 35, the leucine (L) residue shared by GETV‐DL/2025 and GETV‐GY/2025 was rare in the reference strains (1/136), whereas the isoleucine (I) residue carried by GETV‐YY/2025 was the major variant (135/136).

**Table 2:** Fourteen polymorphic amino acid positions in Nsp1, Nsp3, Cap, and E2 among the three GETV isolates and 136 reference strains.

Protein	Position^a^	GETV‐DL/2025	GETV‐YY/2025	GETV‐GY/2025	Consistency^b^	Reference strains (*n* = 136)
Nsp1	298	K	K	Q^c^	No	All K
Nsp1	306	H	Y	H	No	1 Y, 135 H
Nsp1	427	I	T	T	No	31 I, 105 T
Nsp1	473	A	T^c^	A	No	All A
Nsp1	475	T	T	T	Yes	34 T, 102 others^d^
Nsp3	62	S^c^	G	G	No	All G
Nsp3	361	I	I	I	Yes	12 I, 124 others^d^
Nsp3	391	V	V	V	Yes	6 V, 130 E
Nsp3	410	E	E	E	Yes	5 E, 131 others^d^
Nsp3	438	T	T	T	Yes	11 T, 125 others^d^
Nsp3	443	S	S	S	Yes	25 S, 111 others^d^
Nsp3	447	A	V^c^	A	No	1 T, 135 A
Cap	35	L	I	L	No	1 L, 135 I
E2	51	A	A	T	No	1 T, 135 A

^a^Amino acid positions are numbered according to the GETV‐DL/2025 sequence.

^b^“Consistency” indicates whether all three isolates share the same amino acid.

^c^“Unique” refers to an amino acid that is absent from all 136 reference strains.

^d^“Others” refers to amino acids that are not the one specified in the “Reference strains” column. The exact composition is provided in Supporting Information 2: Table S1.

### 3.3. Pathogenicity Analysis of the GETV‐YY/2025 Strain in Piglets

**Figure 3:**
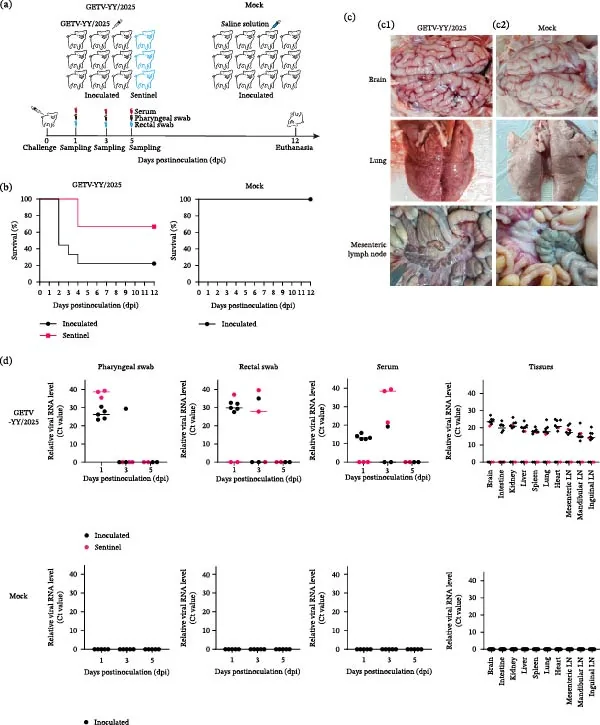
Pathogenicity analysis of the GETV‐YY/2025 isolate in piglets. (a) Schematic diagram of the animal experiment design. (b) Survival curves of piglets. (c) Representative gross pathological lesions in the brain, lungs, and mesenteric lymph nodes from a piglet that died postinfection (c1) and a mock‐infected control piglet (c2). (d) Replication dynamics of GETV‐YY/2025 in piglets. Viral RNA levels were detected by qRT‐PCR and are presented as Ct values, where lower Ct values correspond to higher viral loads; negative samples were assigned a value of 0. Data are shown for pharyngeal swabs, rectal swabs, and serum collected at 1, 3, and 5 dpi, as well as for the indicated tissues collected from piglets that either died or were euthanized at 12 dpi. Prior to RNA extraction, all tissue samples were normalized to 200 mg, and swab or serum samples to 200 μL. Each dot represents an individual sample, and horizontal bars indicate the mean. “LN” denotes lymph node.

Given the high nt identity and similar replication kinetics observed among the three GETV isolates, the GETV‐YY/2025 strain was selected for subsequent pathogenicity evaluation in piglets. Seven‐day‐old piglets were divided into a challenge group and a mock control group, with 12 piglets per group. In the challenge group, nine piglets were i.m. inoculated with 2 × 10^6^·^2^ TCID_50_ of GETV‐YY/2025, while the remaining three piglets served as unchallenged in‐contact sentinels. Mock‐control piglets received an equal volume of sterile saline (Figure 3a). Among the challenge group, inoculated animals exhibited severe clinical signs, including lethargy and diarrhea, which progressed to ataxia, hindlimb paralysis, recumbency, and paddling movements. Mortality reached 77.8% (7/9) in inoculated piglets by the experimental endpoint (12 dpi). Furthermore, one sentinel piglet housed in the same group died at 4 dpi, resulting in a contact transmission mortality rate of 33.3% (1/3). In contrast, no deaths occurred in the mock control group throughout the entire observation period (Figure 3b).

Piglets that died from infection were necropsied immediately, whereas surviving animals were euthanized and examined at 12 dpi. Gross pathological examination of fatal cases revealed cerebral vascular congestion, pulmonary hemorrhagic lesions, and swollen mesenteric lymph nodes. In contrast, tissues from the mock control group appeared morphologically normal (Figure 3c). Viral shedding dynamics and viremia were assessed by quantifying viral RNA levels in pharyngeal swabs, rectal swabs, and serum samples collected at 1, 3, and 5 dpi. Among the challenge group, inoculated piglets showed high viral loads across all sample types as early as 1 dpi, with mean Ct values of 26.4 ± 2.9 in pharyngeal swabs (*n* = 5), 30.3 ± 2.1 in rectal swabs (*n* = 5), and 13.6 ± 1.4 in serum (*n* = 5) (Figure 3d). One of the three sentinel piglets in this group also tested highly positive in the rectal swab and serum at 3 dpi and died the following day (Figure 3b,d). Viral RNA loads were also quantified in various organs, including the brain, intestines, kidneys, liver, lungs, heart, mesenteric lymph nodes, mandibular lymph nodes, and inguinal lymph nodes. Samples were collected immediately upon death in fatal cases or at the experimental endpoint (12 dpi) from surviving animals. In piglets that succumbed to infection, high viral loads were detected in all examined tissues. The highest mean viral load was observed in the inguinal lymph nodes (mean Ct value: 15.9 ± 2.7, *n* = 6). In contrast, no GETV RNA was detectable in any samples from the mock control group (Figure 3d). Taken together, these results demonstrate that the GETV‐YY/2025 strain is highly virulent in piglets, causing severe neurological signs, high mortality, and marked hemorrhagic lesions in the lungs and brain. The infection is characterized by high viremia, efficient viral shedding in rectal and pharyngeal swabs as early as 1 dpi, broad tissue tropism, and efficient contact transmission to sentinel piglets housed in the same pen.

## 4. Discussion

In August 2025, GETV outbreaks occurred on three commercial pig farms in Guangdong and Hunan Provinces, China, resulting in substantial economic losses. Through differential diagnosis and subsequent pathogenicity studies, we confirmed GETV as the causative agent of these outbreaks. In recent years, GETV has shown increasing prevalence in China, posing a growing threat to the swine industry [12, 14, 22, 26–29]. GETV infection is associated with reproductive disorders in sows, including abortion and stillbirth, as well as severe clinical manifestations in piglets, such as diarrhea, neurological signs, and death. Because these manifestations overlap with those caused by PEDV [30], PRRSV [31], and pseudorabies virus [32], an accurate differential diagnosis is essential. Our findings further underscore the necessity of incorporating GETV into routine diagnostic and surveillance programs on commercial pig farms.

To trace the origin of the outbreak strains, we performed phylogenetic analysis. GETV is known to comprise four major lineages (groups I–IV), among which group Ⅲ has become the predominant circulating lineage [16]. With more than 120 group Ⅲ strains reported to date, further subgroup division is required to better elucidate the genetic diversity and evolutionary dynamics of contemporary GETV strains. In the present study, group Ⅲ strains were classified into three subgroups (GⅢ‐a, GⅢ‐b, and GⅢ‐c) based on the phylogenetic topology, bootstrap support values, and pairwise genetic distances of the *E2* gene. This classification is consistent with that proposed by Guo et al. [27], supporting its reliability. Subgroup GⅢ‐a mainly comprises early mosquito‐ and equine‐derived strains; subgroup GⅢ‐b includes bovine‐, fox‐, and swine‐originated strains from China and Japan; and subgroup GⅢ‐c encompasses the majority of group Ⅲ strains isolated from diverse hosts and geographic regions across East Asia. Whether the three subgroups differ in biological properties such as pathogenicity or host adaptation warrants further investigation. A recent work by Sun et al. [21] identified a distinct branch within group Ⅲ, designated as the GETV group Ⅲ variant, characterized by unique amino acid substitutions in E2 and Nsp3 and enhanced virulence in neonatal mice and piglets. However, our *E2* gene‐based analysis showed that the mean genetic distance within this branch was comparable to that among other members of subgroup GⅢ‐c, indicating that its divergence did not reach the subgroup‐level threshold. Although this branch forms a distinct phylogenetic cluster and harbors unique molecular features, we retain it as a lineage within subgroup GⅢ‐c rather than an independent subgroup in order to avoid oversplitting and maintain a practical classification framework.

The three isolates characterized in this study clustered within subgroup GⅢ‐c and shared high nt identity with each other, as well as with the swine‐origin strain GETV‐JX‐CHN‐22‐P7 from Jiangxi Province. The close genetic relationship among these isolates implies possible cross‐regional transmission via mosquito vectors or through the movement of swine or vehicles. Notably, GETV has recently been detected as a contaminant in live PRRSV vaccines [33–35], which may represent an additional route for its spread. However, the precise origin of these outbreak strains requires further investigation.

In this study, comparative amino acid sequence analysis revealed that most GETV proteins are highly conserved, whereas Nsp1, Nsp3, Cap, and E2 harbor a cluster of polymorphisms. Among these, four substitutions (Nsp1 K298Q, Nsp1 A473T, Nsp3 G62S, and Nsp3 A447V) were absent from all 136 reference strains examined, representing novel variants in this dataset that may serve as molecular markers for distinguishing the three isolates. To gain insights into their potential functional relevance, we mapped these residues onto the known domain architectures of alphavirus nonstructural proteins. In Nsp1, the K298Q substitution within the ring‐aperture membrane‐binding and oligomerization domain is implicated in anchoring the replication complex to cellular membranes [19], while the A473T substitution lies in the C‐terminal disordered region, which may influence protein–protein interactions or structural stability [19]. In Nsp3, the G62S substitution is located in the macrodomain, a region involved in ADP‐ribose binding and de‐ADP‐ribosylation activity that is critical for viral replication and host interferon antagonism in other alphaviruses [20]; the A447V substitution falls within the hypervariable domain, which mediates host‐specific interactions and has been linked to viral adaptation and pathogenicity [20]. Although the functional consequences of these specific changes in GETV require experimental validation, their locations within functionally important domains suggest plausible effects on viral replication or immune evasion. More importantly, six positions—Nsp1 T475, Nsp3 I361, Nsp3 V391, Nsp3 E410, Nsp3 T438, and Nsp3 S443—were identical among the three isolates and also highly conserved in a subset of reference strains, including GETV‐JL, GETV‐JX‐CHN‐22‐P7, GETV‐HD, HNzmd‐BY1, and GETV‐CX7. These strains, together with our three isolates, formed a distinct subclade in the phylogenetic tree (Figure 2b). Although these residues were sporadically present elsewhere in the tree, they were largely concentrated within this subclade, suggesting that they may serve as subclade‐associated signatures. Notably, several of these conserved residues (e.g., Nsp3 I361, V391, E410, and T438) are located in the hypervariable domain of Nsp3, where they may influence protein–protein interactions or host factor recruitment [20].

At divergent positions, most reference strains share the residue found in two of the isolates, while the third isolate carries a rare variant—except at Cap position 35, where the residue L shared by the two isolates is itself rare, and the third isolate instead carries the dominant residue I. These patterns imply that the rare variants likely emerged independently and have not become fixed in the population. Nevertheless, these functional predictions should be interpreted cautiously and warrant further validation through reverse genetics—for example, by generating mutant viruses harboring the identified substitutions and assessing their replication, cytopathogenicity, and host innate immune responses both in vitro and in vivo. It is also worth noting that although the reference set used in this study is relatively large, it may not fully represent the global genetic diversity of GETV; thus, the absence of these substitutions from the reference strains does not necessarily confirm their absolute uniqueness. Despite these uncertainties, the present data offer valuable molecular markers for the epidemiological surveillance and phylogenetic classification of emerging GETV strains.

Recent years have witnessed the emergence of multiple highly virulent group III GETV variants across various regions of China. Sun et al. [21] reported several virulence‐enhanced variants in Henan Province that caused 100% mortality in piglets, with specific mutations identified in Nsp3 and E2. Wu et al. [23] isolated a highly virulent strain (GETV‐QJ) in Shanxi Province, also resulting in 100% piglet mortality and associated with severe diarrhea, fever, and intestinal and lung damage, along with several differential substitutions in the E2 protein compared with the type I strain MM2021. Chu et al. [12] identified a novel variant (GDHYLC23) in Guangdong Province, characterized by a unique 32‐nt insertion in the 3^′^ noncoding region and linked to severe piglet mortality and sow reproductive disorders. Notably, GETV‐JX‐CHN‐22‐P7, which belongs to the same GIII‐c subclade as our isolates, has also been reported to cause high mortality in piglets [22]. Although the GIII‐c subclade identified in our study and these previously reported variants all fall within group III and are associated with high virulence, they differ in their specific molecular markers and geographic origins—suggesting that multiple group III sublineages with distinct genetic signatures may be cocirculating in China. In our pathogenicity assessment, infected piglets exhibited characteristic clinical signs and gross pathological lesions, with high viral RNA loads detected in multiple organs and serum, indicative of systemic dissemination. The detection of viral RNA in rectal and oropharyngeal swabs suggests potential viral shedding via fecal and oral‐pharyngeal routes. Notably, our study provides the first experimental evidence that GETV can be transmitted through direct contact, as demonstrated by the fatal infection in one of three cohoused sentinel piglets. This finding highlights the critical importance of rapid isolation of affected animals and stringent disinfection measures during GETV outbreaks.

We acknowledge several limitations in this study. First, the association between the identified amino acid substitutions and the observed high virulence remains correlative, as the functional significance of these mutations has not been experimentally verified. Second, although we demonstrated efficient contact transmission, the sample size of sentinel piglets was small. Third, our pathogenicity evaluation was performed with a single isolate (GETV‐YY/2025), and the virulence of the other two isolates was not directly compared; further studies with additional isolates are warranted. Fourth, the qRT‐PCR method used for viral load quantification detects viral RNA rather than infectious virus, which may not fully reflect the presence of viable viruses in certain samples. Future studies employing reverse genetics, larger animal cohorts, and extended field surveillance will be necessary to address these questions.

## 5. Conclusion

In summary, this study identifies a highly virulent and transmissible GETV subgroup, GⅢ‐c, circulating in commercial pig farms across China. The three isolates carry novel amino acid substitutions in the reference datasets and form a phylogenetically distinct subclade. The representative strain induced severe clinical disease, high mortality, and efficient contact transmission in neonatal piglets. These findings highlight the emerging threat posed by this evolving pathogen to the global swine industry, underscoring the urgent need for enhanced surveillance and the immediate implementation of effective prevention and control measures.

## Author Contributions

**Zhaoying Xiang**: conceptualization, data curation, formal analysis, investigation, methodology, visualization, writing – original draft. **Xingchen Wang**: conceptualization, data curation, investigation, methodology. **Yuchen Huang**: investigation, data curation. **Xinyu Gao:** investigation, data curation. **Lei Zhou, Xinna Ge, and Jun Han:** formal analysis. **Yongning Zhang**: conceptualization, data curation, formal analysis, writing – review and editing. **Hanchun Yang**: funding acquisition, resources, supervision.

## Funding

This study was supported by China Agriculture Research System of MOF and MARA (Grant CARS‐34) and the 2115 Talent Development Program of China Agricultural University.

## Ethics Statement

All animal experiments were conducted in accordance with the animal welfare guidelines issued by the Ministry of Science and Technology of the People’s Republic of China and the National Standard Laboratory Animal—Requirements of Environment and Housing Facilities (GB 14925‐2010). All experimental procedures involving animals were approved by the Laboratory Animal Ethical Committee of China Agricultural University (Approval Number AW40215202‐2‐08).

## Conflicts of Interest

The authors declare no conflicts of interest.

## Supporting Information

Additional supporting information can be found online in the Supporting Information section.

## Supporting information


**Supporting Information 1** Table S2: Pairwise comparisons of viral growth kinetics among the three GETV isolates at each time point using Tukey’s HSD test.

**Supporting Information 2** Table S1: Detailed information on fourteen polymorphic amino acid positions in Nsp1, Nsp3, Cap, and E2 among the three GETV isolates and 136 reference strains.

## Data Availability

The data supporting the findings of this study are openly available in Zenodo at https://doi.org/10.5281/zenodo.20568795 [36].
